# Vitiligo may be associated with an increased genetic risk of cardiovascular disease: A Mendelian randomization study

**DOI:** 10.1016/j.jdin.2024.07.010

**Published:** 2024-08-12

**Authors:** Austin J. Piontkowski, Celina Dubin, Ross O’Hagan, Jeremy Orloff, Camille M. Powers, Nicholas Gulati

**Affiliations:** Department of Dermatology, Icahn School of Medicine at Mount Sinai, New York, New York

**Keywords:** cardiovascular disease, clinical research, general dermatology, medical dermatology, Mendelian randomization, vitiligo

*To the Editor:* Vitiligo is an inflammatory disease that causes patchy depigmentation of the skin. Recent findings suggest a link between vitiligo and cardiovascular disease (CVD), although the existence of a genetic and causal relationship remains unknown. This study seeks to explore the relationship between vitiligo and CVD through the application of Mendelian randomization (MR). Utilizing data from genome-wide association studies (GWAS), MR leverages genetic variation to estimate the causal effects of an exposure on an outcome.^1^ The strategic use of genetic variants effectively eliminates confounding bias, enabling a robust analysis of the causal effects that vitiligo may have on CVD.

GWAS summary statistics were obtained for vitiligo from the GWAS Catalog, a collaborative effort by the National Human Genome Research Institute and European Bioinformatics Institute. This dataset included 8,966,111 single-nucleotide polymorphisms (SNPs) derived from 4680 vitiligo patients and 39,586 controls. Summary statistics for CVD were obtained from the Integrative Epidemiology Unit Open GWAS Project, including 16,380,466 SNPs among 101,866 cases and 116,926 controls. An MR analysis was then performed using the inverse-variance weighted and weighted median methods. Genes associated with SNPs exhibiting an odds ratio (OR) >1.00 were subsequently included in an enrichment analysis. All analyses took place within R version 4.3.1 using the packages “TwoSampleMR” and “enrichR” (Supplementary Methods, available via Mendeley at https://doi.org/10.17632/984w22jmcy.1).

Overall, MR revealed a significant association between vitiligo and CVD with both the inverse-variance weighted method (OR = 1.03, β = 0.029, *P* = .014) and the weighted median method (OR = 1.02, β = 0.021, *P* = .047). There was no evidence to suggest the presence of horizontal pleiotropy (MR Egger *P* = .323). Five significant SNPs (OR >1) were identified, namely: (1) rs10774624, a single nucleotide variation (SNV) of *LINC02356*; (2) rs2476601, a SNV of *PTPN22*, linked to autoimmune conditions; (3) rs12421615, a SNV of *PLCB3*, encoding phospholipase C beta-3; (4) rs1043101, a SNV of *SLC1A2*, involved in glutamate clearance; and (5) rs2111485, a SNV of *IFIH1*, involved in innate immunity (Fig 1). Enrichment analysis further revealed several key pathways involved in the regulation of proinflammatory cytokines, B-cell receptor signaling, and natural killer cells (Fig 2).

**Fig 1:**
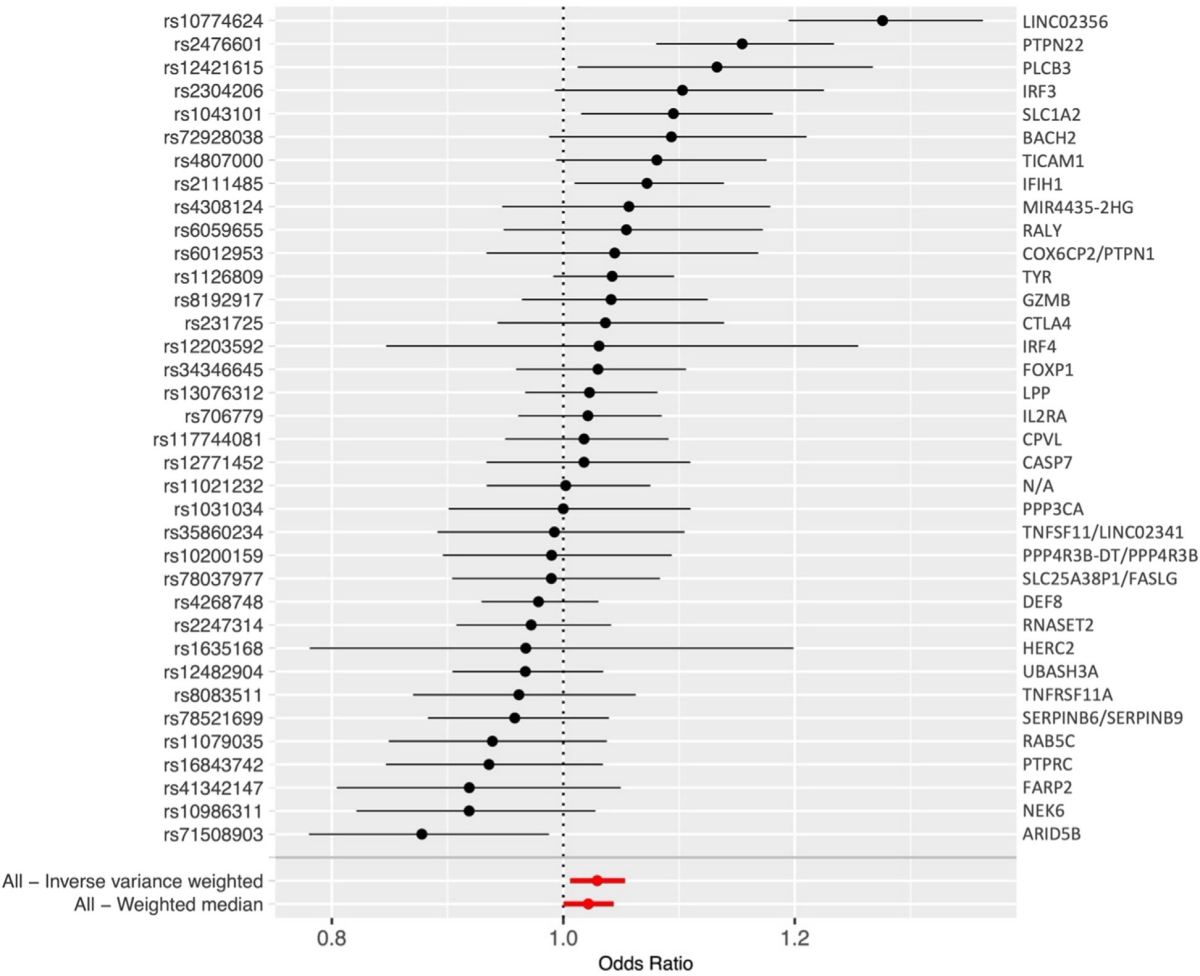
Forest plot depicting the odds of cardiovascular disease for individual single-nucleotide polymorphisms (SNPs) (*left axis*) and corresponding genes (*right axis*).

**Fig 2:**
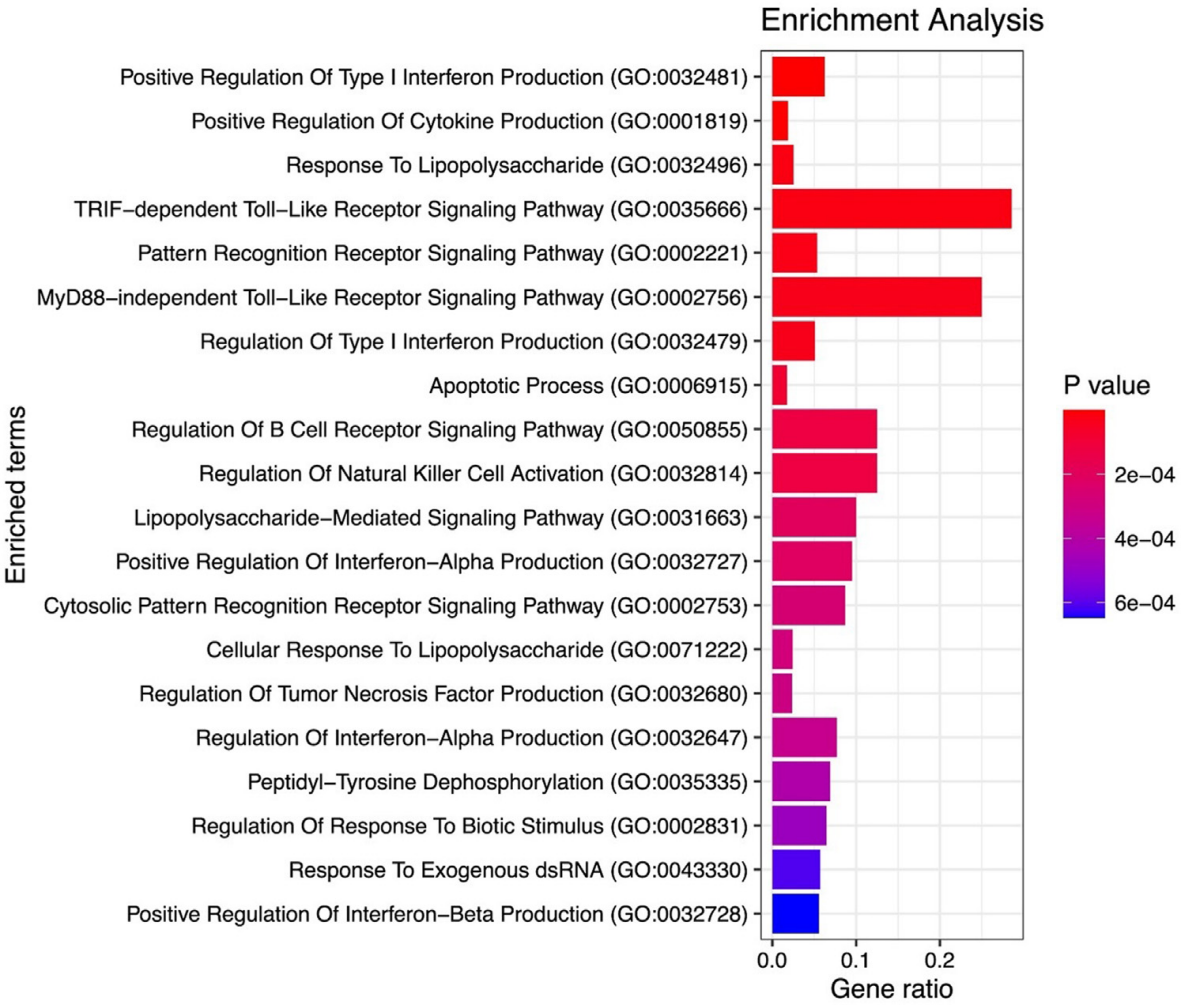
Enrichment analysis of genes corresponding to single-nucleotide polymorphisms (SNPs) with an odds ratio greater than 1.00 utilizing the Gene Ontology Cellular Component 2023 library within the Enrichr database.

This study demonstrates, to our knowledge, the first causal relationship between vitiligo and CVD. Recent research has demonstrated increased rates of hypercholesterolemia and atherosclerosis in vitiligo patients, postulating that the proinflammatory mediators implicated in vitiligo, such as tumor necrosis factor-alpha, interleukin-1, and interleukin-6, also contribute to the development of CVD.^2^, ^3^, ^4^ Our findings align with this hypothesis, as the SNPs and enriched pathways driving this relationship are primarily involved in cell signaling, immunity, and inflammation. However, the use of MR in parameter estimation is often constrained in clinical settings.^5^ Furthermore, the modest magnitude of causal estimates observed in this study may be indicative of compounded estimates that are not representative of the primary SNPs influencing this relationship and the existence of other explanatory variables. Nonetheless, these results unveil a clear genetic predisposition to CVD among vitiligo patients. Consequently, there is a need for heightened surveillance and additional research into preventative care strategies for these patients, as well as risk stratification by racial and ethnic backgrounds.

## Conflicts of interest

None disclosed.
